# Wourara in Tetanus

**Published:** 1860-01

**Authors:** 


*Wourara in Tetanus.*—Years ago, when Mr. Waterton, the South American traveler, known to all children as the man that rode the alligator, was busy in hunting up the wonders of Guiana, much interest was excited in the peculiar poison with which the aborigines of that region tipped their arrows. All the journals, scientific and literary, rung with the wonders of wourali, as it was then called. Its deadly and rapid operation, and its peculiar paralyzing influence upon the spinal cord were observed and commented on. Cases of tetanus in the lower animals were treated with this poison, and its power as an antidote, were fully recognized by the eminent British surgeons who made the observations.

As is often the case with new agents, often attracting great attention for a while, is gradually faded from the notice of the profession, and little or nothing was said of it, except some general remarks upon its activity as a poison. Of late, the interest in the subject has been revived in consequence of the repetition of the old experiments by the French physiologists and surgeons.

It has been proposed to use it in the treatment of tetanus, and several cases have been so treated both in Great Britain and on the continent. Three cases of chronic tetanus have been reported as getting well, after the administration of the poison. The acute cases, however, have all proved fatal. In the last case, under the care of Mr. Follin, of the Hospital Necker, at

Paris, considerable quantities of a solution of the poison were injected into the cellular tissue every half hour. The result was slightly to relax the spasm of the nerve seter muscles, but nothing more. It has now been proposed to use the alkaloid *curaina,* which is the active principle of the poison, in the hope of having a more energetic and more manageable poison. Whatever is done in this direction, it will probably be necessary to push the poisoning to the very verge of dissolution, and trust to the chances of restoring the sinking vitality by the employment of artificial respiration.

